# Compressive and Flexural Properties of Ultra-Fine Coal Gangue-Based Geopolymer Gels and Microscopic Mechanism Analysis

**DOI:** 10.3390/gels8030145

**Published:** 2022-02-25

**Authors:** Xiaoyun Yang, Yan Zhang, Cheng Lin

**Affiliations:** 1 College of Energy and Transportation Engineering, Inner Mongolia Agricultural University, Hohhot 010018, China; ycyangxiaoyun@emails.imau.edu.cn; 2 Department of Civil Engineering, University of Victoria, Victoria, BC V8P 5C2, Canada; chenglin918@uvic.ca

**Keywords:** coal gangue, geopolymer gel, compressive strength, flexural strength, microstructural analysis

## Abstract

Geopolymer gel that possesses advantageous features of fast setting, high strength, and good durability is increasingly used in civil engineering, including rapid retrofit projects, roadway, and other construction projects. Furthermore, geopolymer gel is also a green and economical material as it derives from solid wastes. In this study, activators with different sodium silicate modulus and alkali content were used to activate ultrafine coal gangue and slag powder to prepare coal-gangue-based geopolymers with high strength. To study the influence of slag powder content, sodium silicate modulus, and alkali activator content on strength, a two-stage design was adopted. In the first stage, the orthogonal test with three factors and four levels (10–40% slag, 0.4–1.0 modulus, 16–22%) was used to obtain the influence of each factor on the strength and select the design range of the specimen mix ratio with higher strength. In the second stage, based on the orthogonal experiment, the scope was narrowed to continue to find the optimal excitation scheme and the relationship between the influencing factors and strength. Further, mineral compositional, microstructural, functional group and elemental analyses were performed using X-ray diffraction technique, IR infrared diffraction, electron microscope observation and energy spectrum analysis to elucidate the mechanisms of the strength development. The results show that the factors affecting the geopolymer’s strength were in the order of slag content > alkali content > modulus. The optimum dosage of alkali activator was 18–20%, and the sodium silicate modulus was 0.6–0.8, and the compressive and flexural strength could reach above 40 MPa and 5.9 MPa, respectively. The compressive strength and modulus were in a parabolic relationship. Three types of cementing gels (N-A-S-H, C-A-S-H, and C-N-A-S-H) that were characterized with dense structure and high strength were identified from coal gangue and slag powder after alkali excitation.

## 1. Introduction

Geopolymer gel is a type of Si-Al polymer with three-dimensional network structures. It is usually produced by mixing silico-aluminum or silico-aluminum-calcium solid wastes (such as fly ash, slag, coal gangue, red mud, etc.) with alkaline solutions which act as an activator [1,2,3,4]. The geopolymer possesses superior strength and durability as well as low environmental impact [5], which make it the most likely alternative to ordinary Portland cement.

Coal gangue contains many silicon-aluminum materials which can be geopolymerized under the excitation of alkaline agents. Coal gangue is a solid waste generated in the process of coal mining and washing, accounting for approximately 10% to 15% of the total coal output [6]. Global coal production was about 7.4 billion tons, and coal gangue emissions were about 15–70 million tons [7]. The total amount of coal gangue in China has reached 6 billion, with an annual generation of 5–8 million tons [8,9]. Long-term piling of coal gangue will cause various environmental problems and affect the lives of surrounding residents. Coal gangue is used to prepare geopolymers as engineering construction materials, which can consume a large amount of coal gangue, improve the utilization of solid waste, and reduce environmental pollution. However, geopolymers that only use coal gangue as raw materials have low compressive strength and cannot meet the high compressive strength requirements of engineering [10]. Therefore, coal gangue is often combined with other industrial solid wastes such as steel slag, blast furnace slag, and fly ash to produce geopolymers [11,12,13,14]. Blast furnace slag (BFS) is another solid waste byproduct when pig iron is smelted in the blast furnace at 1400–1600 °C. It is mainly composed of veinstone, ash, co-solvent, and other impurities. Water-quenched BFS, also called water slag, is primarily comprised of calcium aluminosilicate glass formed by quenching the BFS in a high-temperature molten state [15]. The BFS-based geopolymers have a higher compressive strength than the coal gangue-based geopolymers. Therefore, many scholars mix coal gangue and slag to prepare geopolymers. Ma H. et al. studied the effect of slag on the strength of coal gangue-based geopolymer samples and found that the compressive strength of coal gangue-based geopolymer samples with 50% slag content increased rate was 55.98% [16]. Zhu H. et al. measured the compressive strength of coal gangue slag concrete and mixed coal gangue and slag as binder with sand to prepare geopolymer concrete with compressive strength of 62.1 MPa at 28 days [17]. Gao X. et al. mixed coal gangue with 60% mineral powder, and the compressive strength of coal-gangue-based geopolymer could reach 51.7 MPa at 28 days [18]. However, there are still some problems in gangue-slag geopolymers, such as (1) mixing BFS in a large proportion will increase the strength but will also increase the cost; (2) the curing process is complicated, such as steam curing, which can only be prepared in the precast site; (3) the high cost due to the large proportion of activator. Therefore, in this study, ultrafine coal gangue powder and BFS powder (dosage ≤ 40%) were selected as raw materials, and activators with different Na_2_SiO_3_ modulus and alkali dosage were selected to prepare a kind of high strength geopolymer under standard curing conditions. Using a combination of orthogonal experiments and traditional methods, 31 sets of different ratio experiments were used to find the best ratio, activator, and strength relationship. The mineral composition, functional groups, microscopic morphology and elements of the geopolymers were analyzed using X-ray diffraction technique, IR infrared diffraction, electron microscope observation and energy spectrum analysis. The outcomes of this study will be to (1) identify the factors influencing the geopolymer strength development, (2) determine the optimum mixing ratio and procedure, and (3) reveal the mechanisms behind the strength development.

## 2. Experimental Section

### 2.1. Materials

Coal gangue and BFS were sourced from Hejin City, Shanxi Province, China where there are 16 proven main mineral deposits, the majority of which are coal stored in the northwestern part of the city. This area is an extension of the Xiangning county coalfield, covering an area of about 68 square kilometers. The geological reserves are about 680 million tons.

#### 2.1.1. Coal Gangue

**Figure 1:**
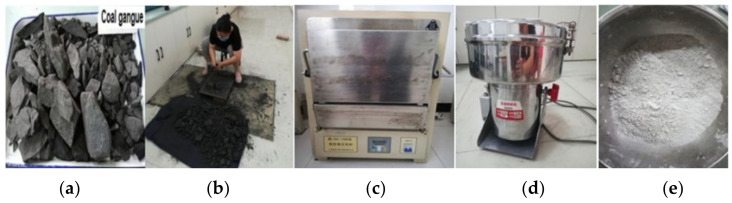
Flow diagram of preparation of UCGP. (**a**) raw coal gangue, (**b**) manual smash, (**c**) calcined 2 h, (**d**) crushed it, (**e**) UCGP.

**Figure 2:**
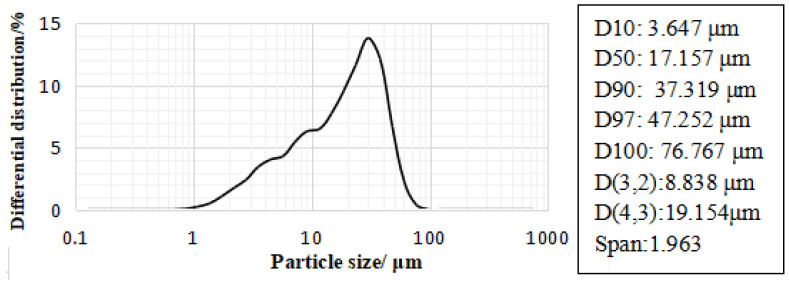
UCGP particle size distribution.

The finer the particle size of the material, the better its activity. Coal gangue particles must be less than 80 μm to have a good excitation effect [19]. In this study, a kind of ultra-fine coal gangue powder (UCGP) was prepared. Figure 1 shows the preparation process of UCGP. Firstly, the gangue was broken into smaller sizes and subsequently calcined in a muffle furnace at 700 °C for 2 h. Finally, a high-speed ore grinder was used to grind the gangue into UCGP. The particle size distribution and chemical composition of UCGP are shown in Figure 2 and Table 1.

#### 2.1.2. Blast Furnace Slag

**Figure 3:**
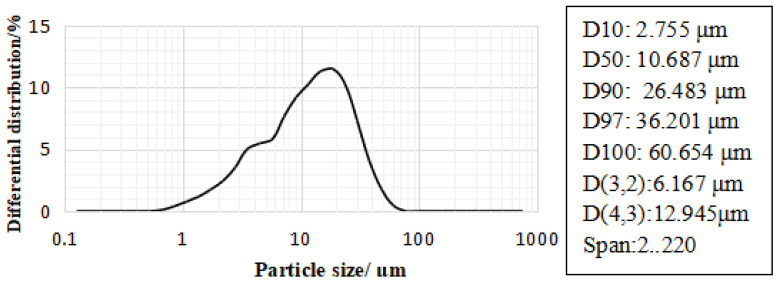
Particle size distribution of BFS.

The blast furnace slag (BFS) used was Grade S95 and its particle size distribution is shown in Figure 3. The basic properties of BFS are summarized in Table 2.

#### 2.1.3. Alkali

Na_2_SiO_3_ is a good activator commonly used in geopolymers. The activation effect is related to the modulus of sodium silicate (modulus is the ratio of SiO_2_ to Na_2_O) and the dosage of alkali. The modulus of a sodium silicate excited geopolymer is usually about 1 [12], and the mixing ratio of the alkali activator is about 20%. The modulus of liquid sodium silicate sold on the market is above 2.25, and too high a modulus of sodium silicate is not conducive to the excitation of geopolymer, so the modulus needs to be reduced. Adding NaOH is a common method to reduce the modulus. Na_2_SiO_3_(liquid) used in the test, purchased from Jiashan Yourui Refractories Co., Ltd., Jiaxing city, China, SiO_2_ content is 29.99%, Na_2_O content is 13.75%, Baume degree is 50 Be, modulus(M) is 2.25. NaOH analytically pure (NaOH ≥ 96%, Yongda reagent, Tianjin city, China), The equations for calculating the modulus are Equations (1) and (2) [20]. The modulus involved in the test ranged from 0.4 to 1.4, and the alkali dosage was set at 16–22%.

(1) $$m_{\mathrm{NaOH}}=\frac{200M_{\mathrm{NaOH}}}{P}\left(\frac{\omega_{{\mathrm{SiO}}_{2}}}{M_{{\mathrm{SiO}}_{2}}\times M_{s}^{\prime }}\right)-\frac{\omega_{{\mathrm{Na}}_{2}O}}{M_{{\mathrm{Na}}_{2}O}}$$

(2) $$m_{H_{2}O}=\frac{m_{\mathrm{NaOH}}\times \omega_{H_{2}O}^{\prime }-\frac{m_{\mathrm{NaOH}}\times M_{H_{2}O}\times P}{2M_{\mathrm{NaOH}}}+100\times \left(\omega_{H_{2}O}^{\prime }-\omega_{H_{2}O}\right)}{1-\omega_{H_{2}O}^{\prime }}$$

where m_NaOH_—quality of solid NaOH. m_H_2_O_—deionized water quality, g. M_SiO_2__—the molar mass of SiO_2_, 60 g/mol. M_Na_2_O_—molar mass of Na_2_O, 62 g/mol. M_NaOH_—molar mass of NaOH, 40 g/mol. M_H_2_O_—molar mass of water, 18 g/mol. $\omega_{H_{2}O}$—mass fraction of H_2_O in raw Na_2_SiO_3_. $\omega_{H_{2}O}^{\prime }$—mass fraction of water in the modified Na_2_SiO_3_. $\omega_{{\mathrm{Na}}_{2}O}$—mass fraction of Na_2_O in the raw Na_2_SiO_3_. $\omega_{{\mathrm{SiO}}_{2}}$—mass fraction of SiO_2_ in raw Na_2_SiO_3_. $M_{s}^{\prime }$—modulus of modified Na_2_SiO_3_. P—purity of solid NaOH, calculated at 96%.

### 2.2. Preparation and Strength Testing of Geopolymers

**Figure 4:**
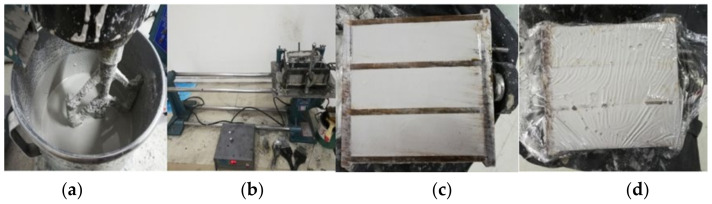

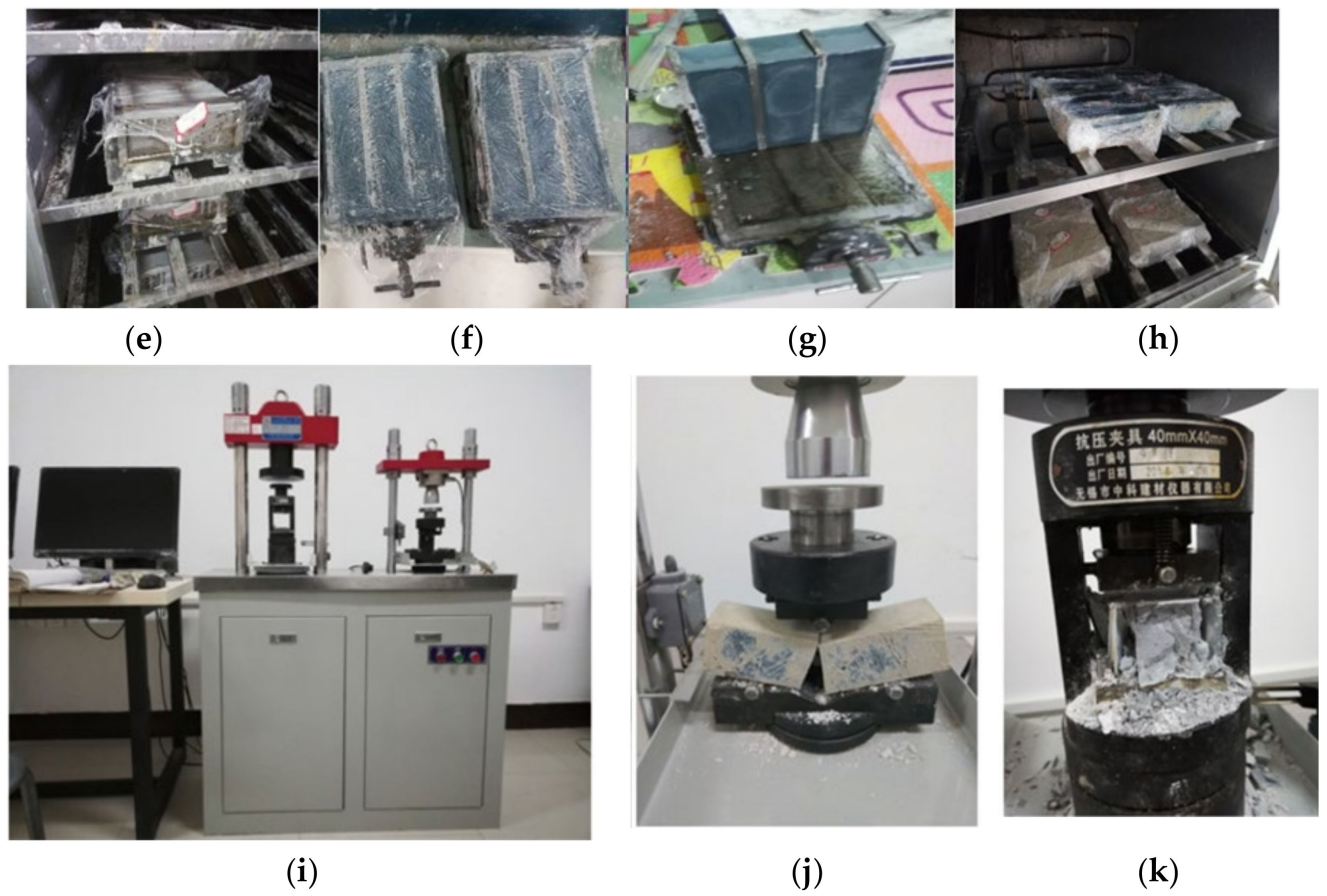
Preparation process and strength test of geopolymer: (**a**) stirring of slurry, (**b**) vibration of the vibrating table, (**c**) complete tapping, (**d**) covering with plastic wrap, (**e**) tank maintenance, (**f**) take out after 24 h, (**g**) demolding, (**h**) continue maintenance, (**i**) automatic compression and flexural testing machine, (**j**) flexural test, (**k**) compression test.

UCGP with good activity was mixed with BFS powder (10%, 20%, 30%, 40%), activated by NaOH + Na_2_SiO_3_ activator (SS activator), with a liquid-binder ratio of 0.45 (Liquid mass = water mass + water mass in liquid Na_2_SiO_3_. Binder mass = UCGP mass + slag mass). A cement paste mixer was used to mix the materials, and the samples were prepared and placed in a standard curing box with constant temperature (20 ± 2 °C) and humidity (>95%). During the curing, the samples were initially wrapped with film for 24 h, which were then demolded and continued curing for 3, 7, 28 days. After the prescribed curing times, the samples were subjected to the compressive strength and flexural strength testing in an automatic compression and flexural tester (WYA-300B automatic flexural and compressive testing machine, Wuxi Xiyi Building Materials Instrument Factory, Wuxi city, China). The test intensity was the average of six test pieces. The preparation process and strength testing test of geopolymers are shown in Figure 4. From the strength tests, those samples with the highest 28-day strength were chosen for the subsequent microstructure analyses.

### 2.3. Characterizations

Geopolymers were ground and soaked in absolute ethanol for 24 h to terminate hydration, and then the dried powders were scanned by X-ray diffractometer (XRD, D8 Advance, Bruker AXS, Karlsruhe, Germany, the scanning angle is 5°–90°, 8° per minute). The results were further processed using a computer program Jade 6.0, from which the mineral compositions were identified. Furthermore, changes in functional groups and chemical bonds were analyzed by FTIR (Fourier Transform infrared spectroscopy, Nicolet iS5, Thermo Fischer Scientific Inc., Waltham, MA, USA). The microscopic morphology, element type and content were obtained by SEM-EDS (Scanning electron microscopy, Zeiss Sigma 500, Zeiss, Oberkochen, Germany. Energy dispersive spectroscopy, Inca250X-Max20, dual detector system, Oxford, UK).

### 2.4. Experimental Design

Because the production of a geopolymer sample consists of many components and sequences, the mixing was carefully designed to identify the optimum mixing ratio and evaluate the influencing factors, which was divided into two stages. Stage I (Table 3 and Table 4) involves the use of a three-factor, four-level orthogonal test to identify the optimum mixing ratio, before determining the level of each factor, to accurately locate its range value, many preliminary experiments were carried out in combination with existing studies. In the preliminary experiments, it was found that the geopolymers with alkali content less than 16% did not have polymerization reaction for a long time (more than 48 h), and it was difficult to be demoded. The geopolymers with alkali content greater than 22% showed many salts’ efflorescence phenomenon. Geopolymers with modulus in the range of 0.4 to 1 can quickly form strength in the early stage, thereby locking the range of alkali content and modulus. Stage II (Table 5) involves evaluating the effects of modulus or alkali content using the sample with the optimum mixing ratio determined in Stage I.

## 3. Strength Test Results

### 3.1. Analysis of Orthogonal Test Results (Stage I)

**Figure 5:**
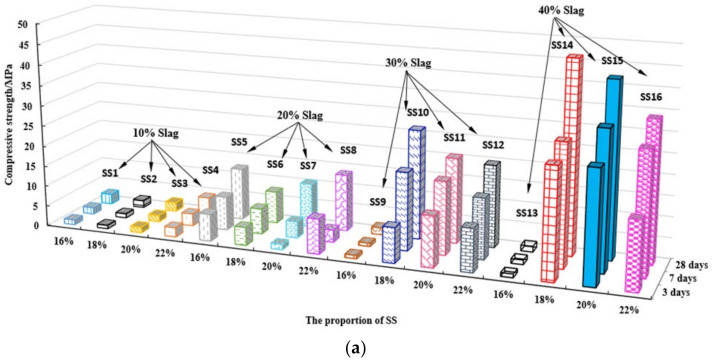

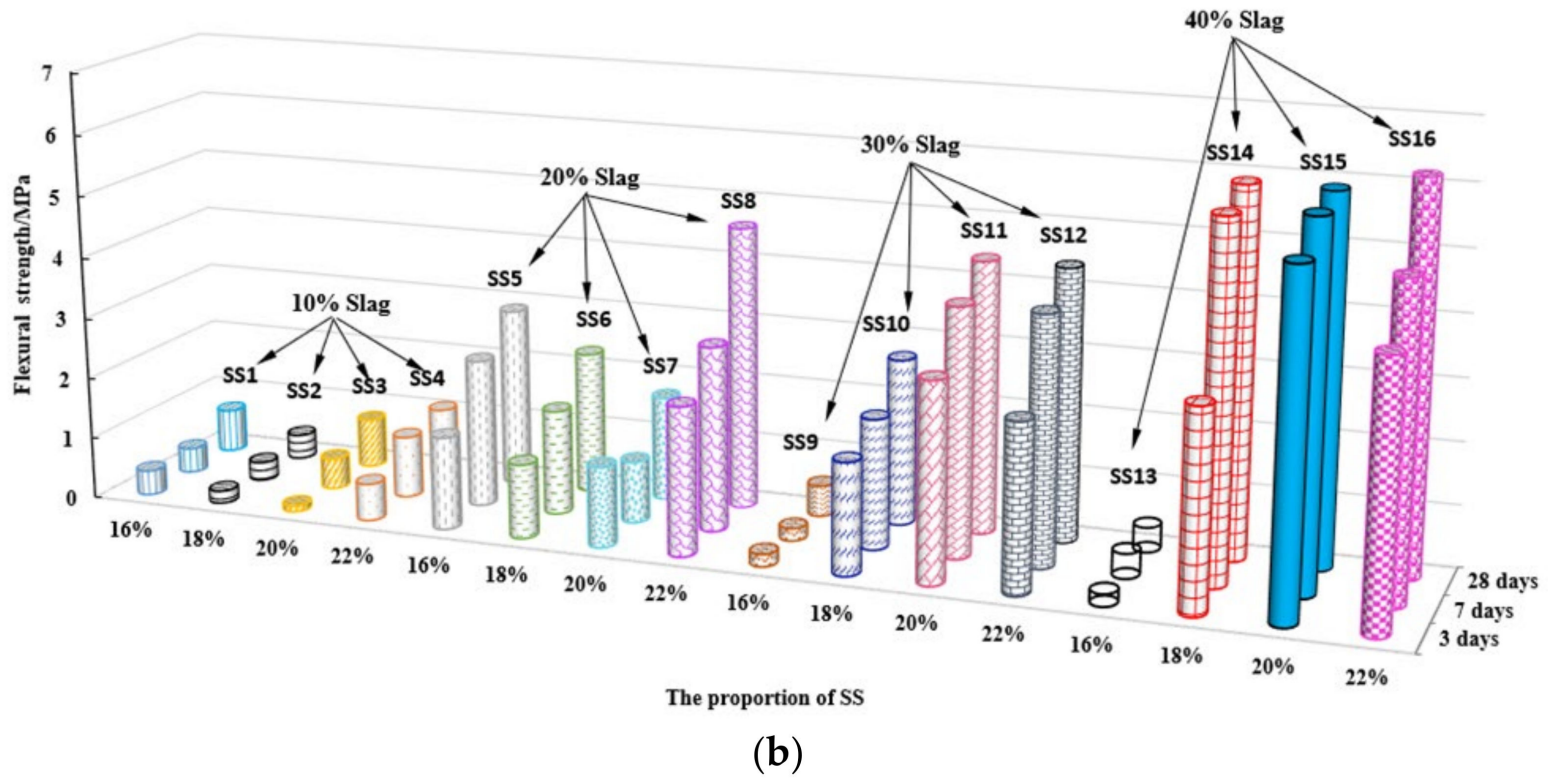
Compressive and flexural diagram of 16 groups of SS activator specimens. (**a**) compressive strength diagram (**b**) flexural strength diagram.

Figure 5 shows the compressive and flexural strength results of 16 groups of SS activator specimens in the orthogonal test (Table 3 and Table 4). It can be seen from Figure 4 that both compressive and flexural strengths increased with the increase in the BFS powder. Samples SS14 and SS15 appeared to have the highest compressive and flexural strengths, e.g., 28-day compressive strength >40 MPa and 28-day flexural strength >5.5 MPa.

To better analyze the orthogonal test data, the expectation and F_ratio_ of the intensity data were calculated, and the results are presented in Table 6. Based on the expectations and F_ratio_ values, the order of each influencing factor can be determined. From the expectations order in the table, the order of the influence strength of each factor is slag content > alkali content > modulus. Comparing the values of each factor F_ratio_ can also determine the ranking of the influencing factors, and the ranking of the influencing factors is consistent with the expectation analysis. The optimum mixing ratio for the short-term (3 and 7 days) compressive strength was A4B3C2 (40% BFS, 20% SS, M0.6), while that for the long-term (28 days) compressive strength was A4B2C2 (40% BFS, 18% SS, M0.6).

**Figure 6:**
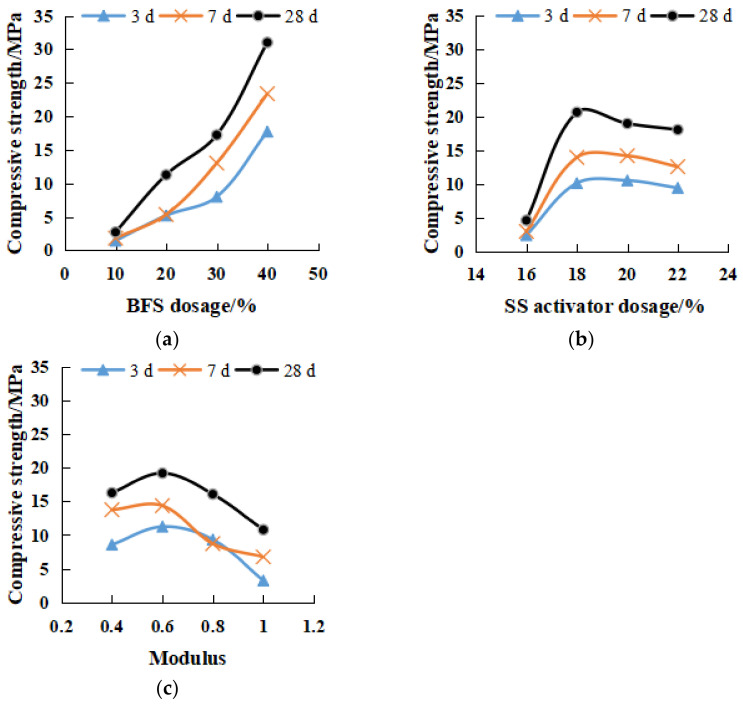
Influence of various factors on compressive strength. (**a**) different dosage of BFS. (**b**) different dosage of SS activator. (**c**) different modulus.

Figure 6 shows the variations of the compressive strength with the influencing factors of BFS content, SS content, and modulus as well as curing times. In general, compressive strength was gained as the curing time increased. Among the other influencing factors, BFS was most beneficial in increasing the compressive strength, showing an exponential incremental relationship (Figure 6a), SS activator showed a limited beneficial effect (Figure 6b), and the modulus showed a least beneficial effect (Figure 6c) on the development of compressive strength. Specifically, the compressive strength for 40% BSF was approximately 11 to 12 times that for 10% BFS. Figure 6b shows that the compressive strength was barely developed when SS content <16%, indicating a low activation level. The activation was rapidly developed and reached the maximum when the dosage reached 18%. A slight attenuation was found when SS addition was 22%, which demonstrated that excessive alkali inhibited the strength development. Figure 6c shows that the optimum modulus was about 0.6, and a further increase in modulus had a negative effect on the strength development.

**Figure 7:**
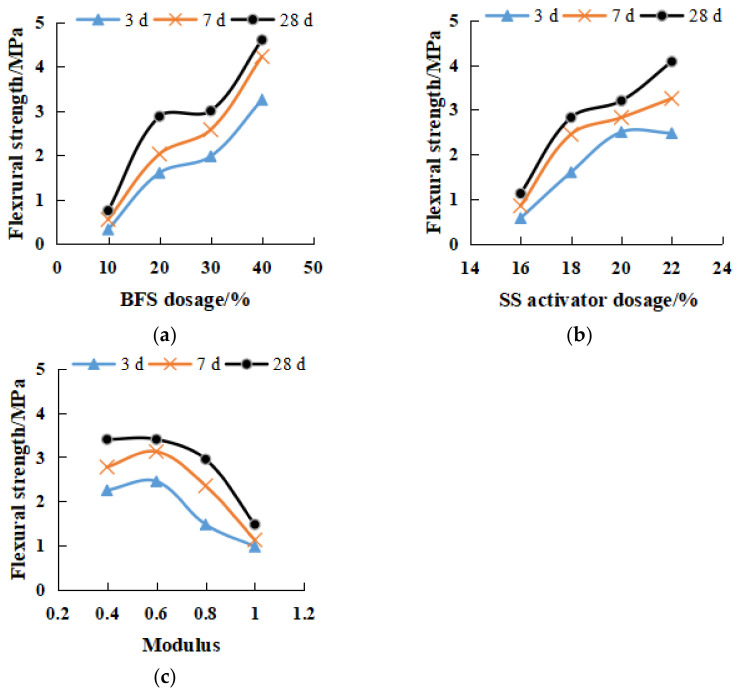
Influence of various factors on flexural strength. (**a**) different dosage of BFS (**b**) different dosage of SS activator (**c**) different modulus.

Figure 7 depicts the effects of various influencing factors on the flexural strength of the geopolymers. Overall, similar trends to those for compressive strength (Figure 6) were observed. Figure 6a indicates that the flexural strength for 40% SS was 6–9 times that for 10% SS. Figure 7b reveals that as opposed to a plateau reached in the compressive strength (Figure 7b, the flexural strength gained continuously with the increase in SS content). Figure 7c also shows the optimum modulus of 0.6 in terms of the flexural strength development, which was agreeable to that observed for the compressive strength.

### 3.2. Analysis of the Influencing Factors (Stage II)

This section is based on the orthogonal experiment (Stage I) and further explores and analyzes the effects of modulus and alkali content on the compressive and flexural strength.

#### 3.2.1. Effects of Modulus on Compressive and Flexural Strength

**Figure 8:**
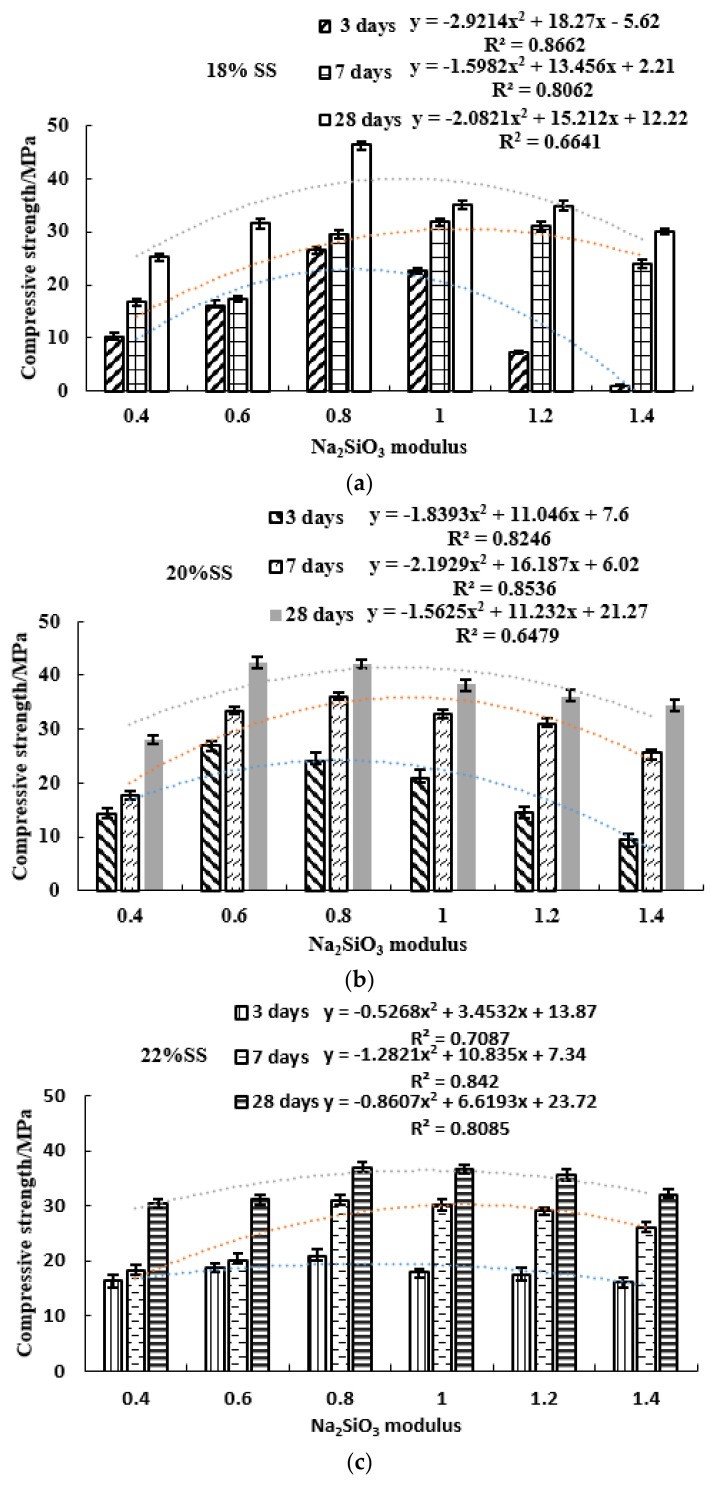
Compressive strength of different modulus geopolymers at 3, 7, 28 days. (**a**) 18% SS (**b**) 20% SS (**c**) 22% SS.

Figure 8 presents the variations of compressive strength with modulus and SS dosage. The 28-day compressive strength reached more than 30 MPa. The maximum compressive strength of 18% SS, 20% SS and 22% SS could reach 46.4 MPa, 42.3 MPa and 37 MPa, respectively. In the same alkali dosage, the relationship between strength and modulus was a quadratic parabola, as shown in Equation (3) The best modulus range was between 0.6 and 0.8, indicating that the ratio of sodium hydroxide and sodium silicate was close, and the two activators could play a good excitation effect together. The modulus was lower or higher than the range value, showing a decreasing trend. As shown in Figure 8a, the alkali content was small, and the compressive strength decreased quickly outside the optimal range; namely the secondary parabola had a small opening, a large coefficient |a| value, and a small coefficient c value. Figure 8c shows a large alkali content. Outside the optimal range, the compressive strength decreased slowly; namely the quadratic parabolic opening is large, the coefficient |a| is small, and the coefficient c value is large. Additionally, the compressive strength of 22% SS was lower than that of 20% SS (Figure 8b), showing the phenomenon of excessive alkali inhibition. It can also be seen from the figures that the curing time was as short as 3 days; the smaller alkali content had a better quadratic fit.

(3) $$y={\mathrm{ax}}^{2}+\mathrm{bx}+c$$

where y is compressive strength, x is Na_2_SiO_3_ modulus. a, b, and c are coefficients.

Table 7 compares the ratio of 3-day and 7-day compressive strength to 28-day compressive strength, respectively. The strength of geopolymers developed rapidly, and the 3-day compressive strength was 50–65% of the 28-day compressive strength (18% SS M0.6–M1, 20% SS M0.4–M1 and 22% SS M0.6–M0.8). According to the requirements of concrete, the 7-day compressive strength must reach more than 70% of the 28-day compressive strength. The ones that meet the requirements are 18% SS M1–1.4, 20% SS M0.6–M1.4, 22% SS M0.8–M1.4. The compressive strength of geopolymers with relatively lower modulus could increase rapidly in 3 days, while the strength of geopolymer with higher modulus increased rapidly in 7 days. Therefore, for emergency engineering (quick road repair, dam repair, bridge repair, etc.), it is recommended to choose the ratio with low modulus to achieve the strength requirements quickly.

**Figure 9:**
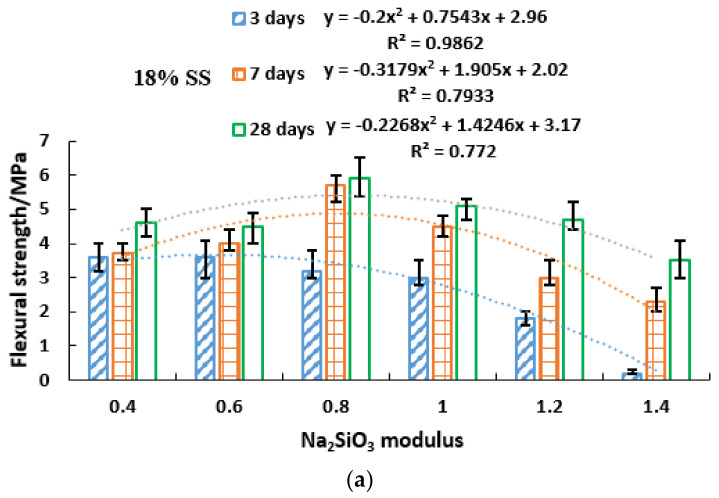

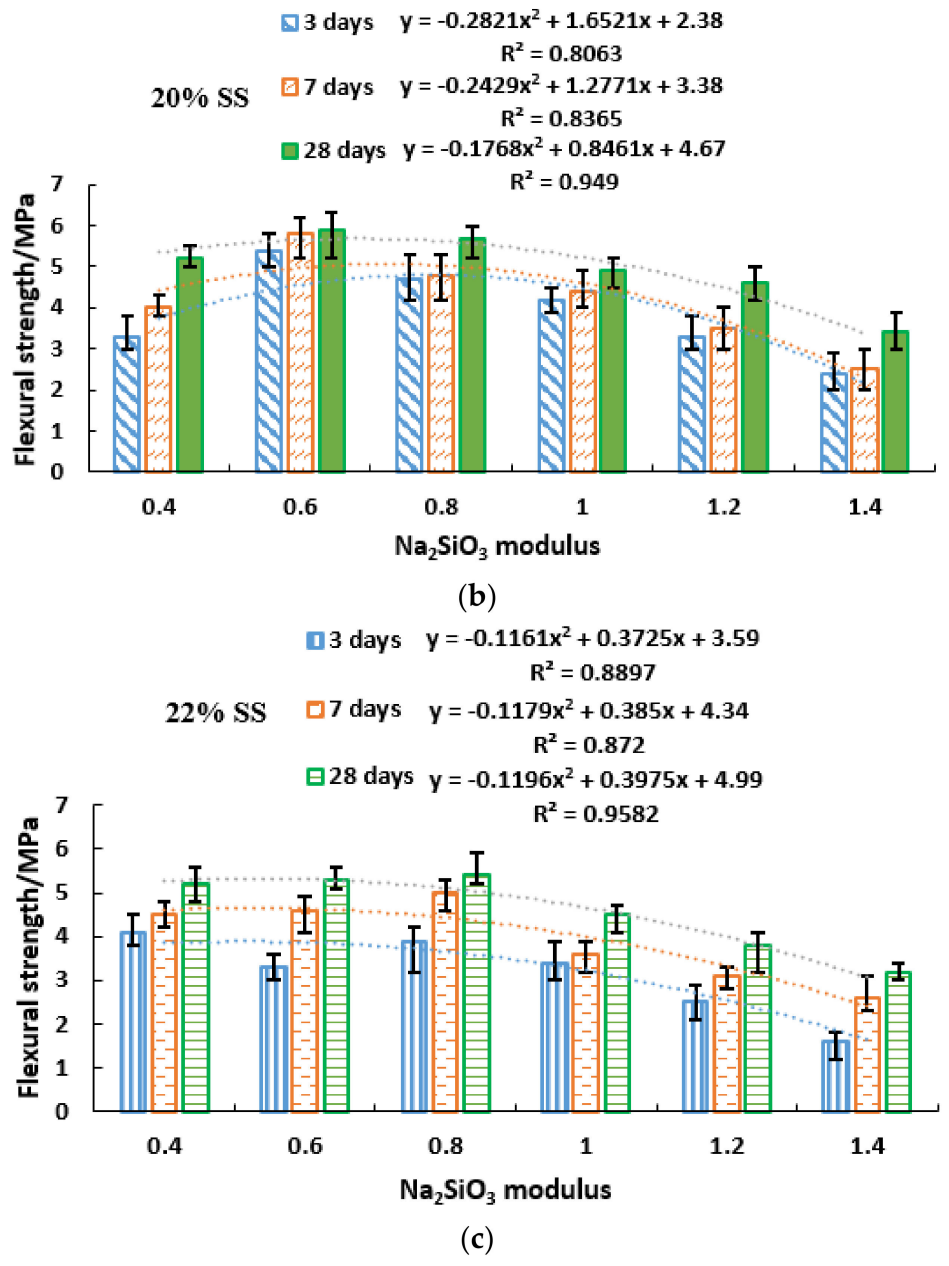
Flexural strength of different modulus geopolymers at 3, 7, 28 days. (**a**) 18% SS (**b**) 20% SS (**c**) 22% SS.

The relationship between geopolymer modulus and flexural strength is shown in Figure 9. The 28-day flexural strength was above 4 MPa, and the maximum flexural strength of 18% SS, 20% SS and 22% SS was 5.9 MPa, 5.9 MPa and 5.4 MPa, respectively. M0.6–M0.8 modulus was the best modulus. Low modulus could form high flexural strength in the early stage. The modulus and flexural strength also reflect the quadratic parabola; 20% SS and 22% SS fit well.

Table 8 shows the ratio of 3-day and 7-day compressive strength to 28-day flexural strength. Except for 18% SS M1.2–1.4, which had lower flexural strength for 3 days and 7 days, the other flexural strengths were relatively high, ranging from 50% to 80% for 3 days, and above 60% for 7 days. The 7-day flexural strength of 18% SS M0.4–M1, 20% SS M0.6–1 and 22% SS could reach more than 80% of 28-day flexural strength. Compared with the compressive strength (Table 7), the growth rate of the flexural strength was higher than that of the compressive strength, and the growth rate of flexural strength of low modulus (M = 0.4–1.0) at both the early and late stages was faster. However, the growth rate of compressive strength of high modulus (M1.0–1.4) at the late day was faster. Therefore, high modulus in the early stage inhibited the strength development, while the compressive strength increased rapidly in the later stage. The low modulus was beneficial to increase the early strength, and the later stage flexural strength increased rapidly.

#### 3.2.2. Effects of SS Alkali Activator Dosage Factor on the Compressive and Flexural Strength

**Figure 10:**
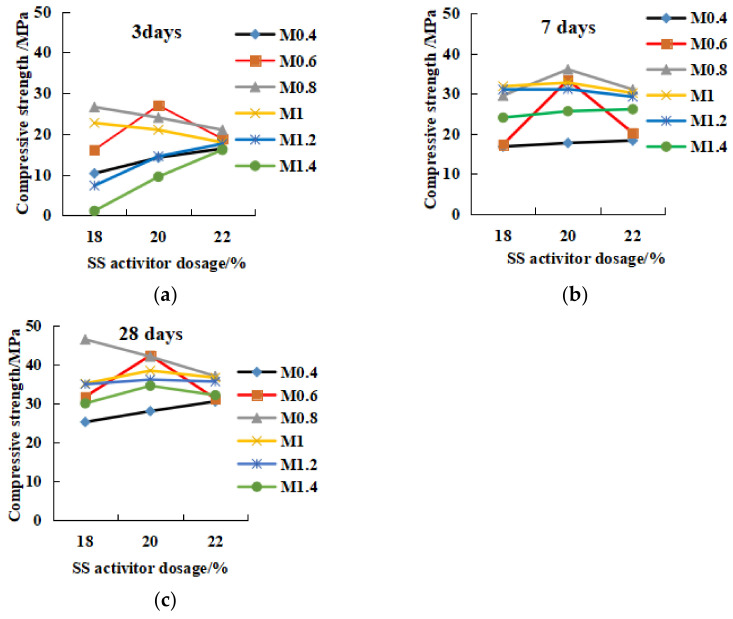
The relationship between the dosage of SS activator and the compressive strength. (**a**) 3 days, (**b**) 7 days, (**c**) 28 days.

**Figure 11:**
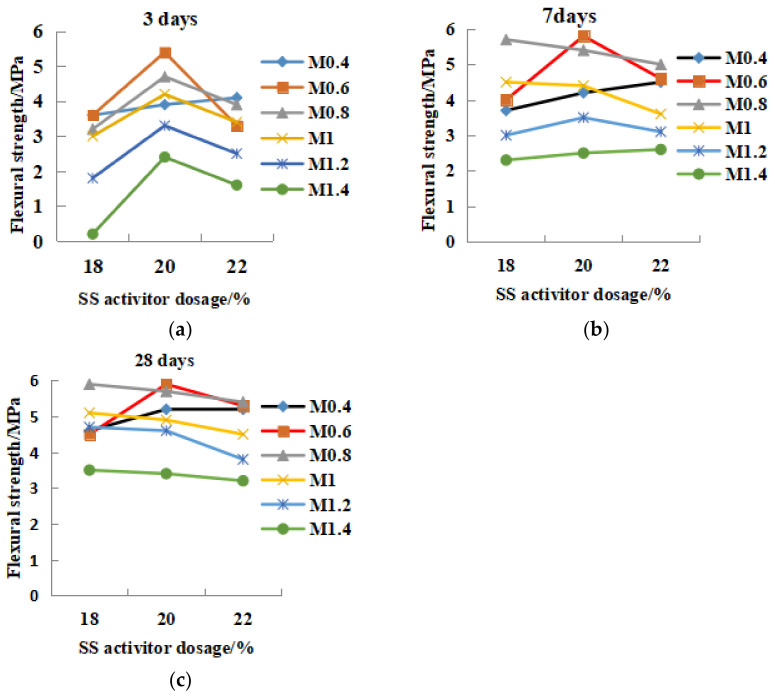
The relationship between the dosage of SS activator and the flexural strength. (**a**) 3 days, (**b**) 7 days, (**c**) 28 days.

Figure 10 and Figure 11 show the relationship between SS alkali content and the compressive and flexural strength. In the 3-day curing period (Figure 10a), high modulus or low modulus (M0.4, M1.2 and M1.4) reflect the increase in compressive strength with the increase of alkali content. Therefore, M ≥ 1.2 or M ≤ 0.4 could increase the initial strength by increasing the alkali content. The strength of M0.8–M1 geopolymer decreased due to the increase of alkali content, which showed the phenomenon of over-alkali inhibition with alkali content greater than 18%. M0.6 20% alkali content was the optimal content, and the strength was reduced outside the 20% range. During the 7-day curing period (Figure 10b), over-alkali (≥22%) in M0.6–M1.2 would inhibit the strength increase. The low-mode and high-mode (M1.4 and M0.4) still showed that alkali increase could increase the strength. In the 28-day period (Figure 10c), except for M1.4, the other modulus all reflected the over-alkali inhibition phenomenon with alkali content ≥ 22%. Therefore, the optimal dosage of alkali excitation should be between 18% and 20%. In Figure 11, for the 3-day flexural strength, except for M0.4, all other geopolymers had 20% alkali content as the optimal content, and less than 20% alkali content increased with the increase in alkali content; more than 20% was over-alkali inhibition. At 7 days, except for M0.4 and M1.4, all others were over-alkali inhibition. After 28 days, in terms of flexural strength, all geopolymers showed over-alkali inhibition. In conclusion, for high modulus and low modulus (M ≥ 1.2, M < 0.6), the early compressive and flexural strength could be improved by increasing the alkali. For 0.6 < M < 1.2, the optimal alkali content was 18–20%. High alkali content would inhibit the strength development, while low alkali had a lower excitation effect.

### 3.3. Compared with OPC

Concrete grade is divided into fourteen grades, namely C15, C20, C25, C30, C35, C40, C45, C50, C55, C60, C65, C70, C75, C80. According to C15, the flexural strength is 3.0 MPa. For every 5 MPa increase in strength, the flexural strength increases by 0.5 MPa. Table 9 describes the comparison of the compressive and flexural strengths of geopolymers and OPC. The highest compressive strength was SS14 (18% alkali, M0.8), which can reach C45, but its flexural strength is 0.1 MPa lower than that of C45, reflecting the characteristics of brittle materials. The flexural and compressive resistance of geopolymers with 20% alkali content and M0.6–0.8 (SS15 and SS23) have reached the C40. The geopolymer that can be matched with the standard C35 is the 18–22% alkali and M0.8, M1 (SS19, SS24, SS28). SS25 (20% alkali, M1.2) and SS30 (22% alkali, M1.2) also achieved C35 strength, with the former showing brittle properties and the latter showing high toughness. The standard C30 is 18% alkali, M0.6 or M1.2 (SS18 and SS20). Additionally, 22% alkali, M0.4–M0.6 (SS16 and SS27) geopolymers exhibit high flexibility, while 18–22% alkali, M1.4 (SS21, SS26 and SS31) exhibit brittle material properties. The geopolymer that meets C25 is 18–20% alkali, M0.4 (SS17 and SS22), of which SS17 has high flexural resistance. In conclusion, geopolymers with higher modulus (large proportion of sodium silicate) reflect the characteristics of brittle materials; low modulus (more sodium hydroxide) reflects high toughness. At 18% alkali content, the strength is rated C30–C35.

## 4. Microstructural Analysis Results

The compressive and flexural strengths of SS14 and SS15 are both above 40 Mpa, Therefore, samples SS14 and SS15 (28 days) after strength tests were further analyzed using XRD, IR, as well as SEM and EDS. Through these tests, the mineral composition, element types and contents, microscopic morphology and internal structure of the samples were compared, and the mechanisms behind the strength developed were discussed.

### 4.1. XRD

Geopolymerization includes many complex stages, such as dissolution, precipitation, recombination, gel, condensation, etc. [21]. As the base materials of the geopolymers were coal gangue and BFS, the hydration products were mainly composed of two categories after being activated by alkali. One is N-A-S-H (natrium aluminosilicate hydrate) geopolymer formed by alkali activated coal gangue [22], and the other is the main crystalline phase of C-A-S-H (calcium aluminosilicate hydrate) formed by alkali activated slag [23]. N-A-S-H has a zeolite-like structure, in which a metal cation such as Na^+^ enters the three-dimensional network to form a zeolite-like structure [24]. C-A-S-H is an amorphous state gel, in which a new phase of Al^3+^ enters the C-S-H structure [25], a part of Al^3+^ replaces Si^4+^ in the silicon-oxygen tetrahedron, and the other part enters the C-S-H layer to render an electrically neural structure and increase the degree of polymerization of the silicon-oxygen tetrahedron [26].

**Figure 12:**
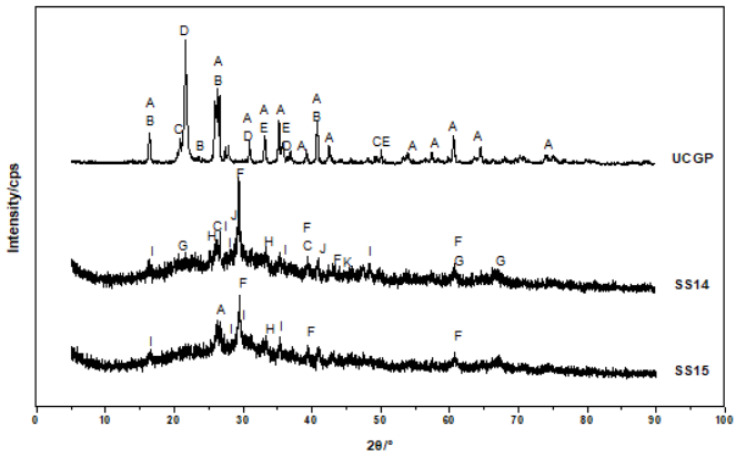
XRD spectrums of SS14, SS15 and UCGP. A. Mullite B. Sillimanite C. Quartz D. Cristobalite E. Hematite F. Calcite G. Nepheline H. Analcime I. Calcium sodium aluminum zeolite J. Gehlenite K. Grossular.

Figure 12 shows XRD spectrums of SS14, SS15 and UCGP. It reveals that the UCGP was mainly composed of mullite, sillimanite, quartz, cristobalite and hematite. After the UCGP was activated by the SS alkali, the main peaks of cristobalite, silica and mullite between 20 and 30° disappeared and decreased in other ranges. The characteristic peak of calcium carbonate appeared at 30°, and other minerals appeared in the form of miscellaneous peaks, mainly nepheline, analcime, calcium sodium aluminum zeolite, gehlenite, grossular. Nepheline and analcime belong to N-A-S-H gel. Gehlenite and grossular are C-A-S-H, calcium sodium aluminum zeolite is C-N-A-S-H gel (calcium natrium aluminum silicate hydrate. The peak strength of all substances in SS14 was higher than that in SS15, indicating that more substances were generated, which corresponds to its higher compressive strength.

### 4.2. FTIR

**Figure 13:**
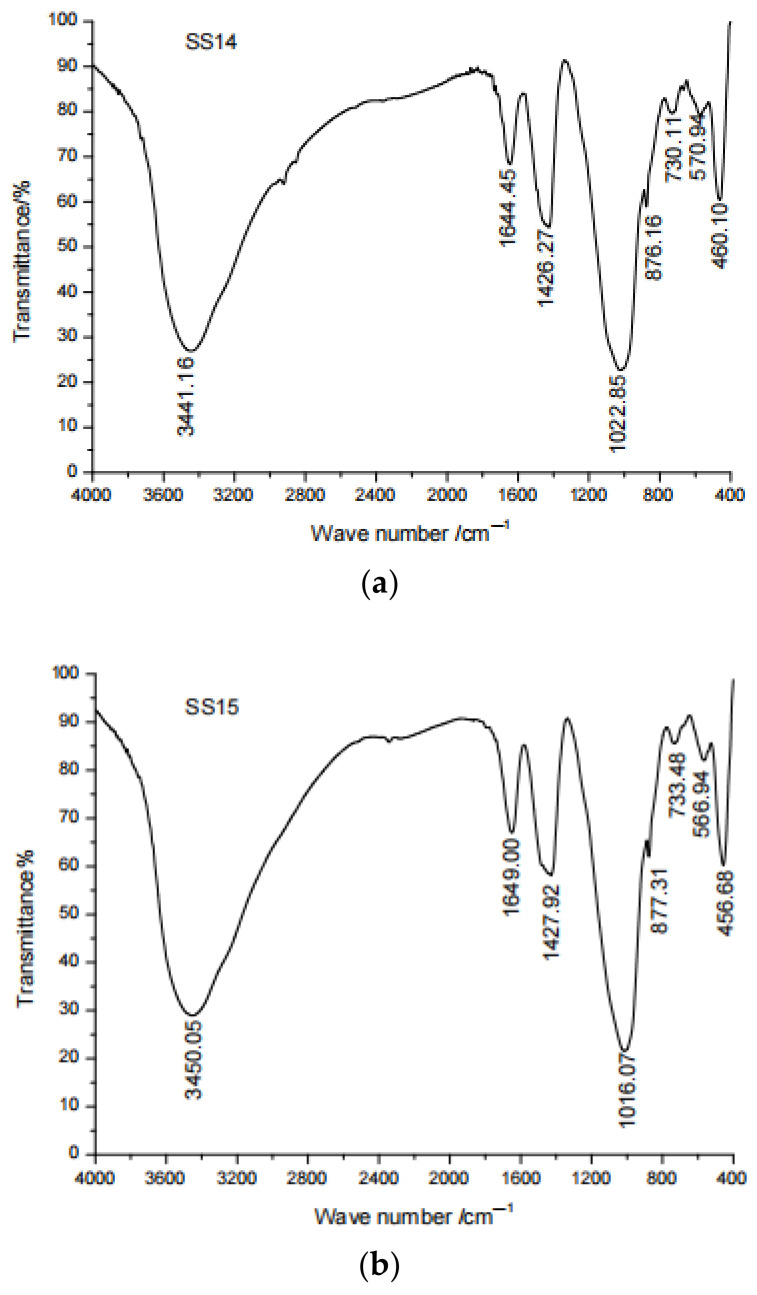
FTIR spectra of geopolymers after 28 days of curing. (**a**) SS14 (**b**) SS15.

Infrared spectroscopy analysis is an important analysis method for characterizing molecular structure without any restriction on samples. It can detect the transformation of molecules and bonds of coal gangue-based geopolymers. Figure 13 shows the IR spectrum of the 28-day geopolymer samples (SS14 and SS15). The main peaks of the geopolymer characteristic spectrum lines appeared virtually at the same positions, indicating that they had similar molecular structures. The Si-O-Si, Al-O-Al and Al-O-Si bonds were broken in a strong alkaline environment, resulting in an increase in the aluminum-silicon monomer during the geopolymerization process [27]. The wave numbers of 3441–3450 cm^−1^ and 1644–1649 cm^−1^ where the peaks were identified corresponded to the stretching vibration of and the bending vibration of the hydroxy group, respectively, which indicated that bound water was produced in the polymerization process [22,28]. Therefore, during the polymerization process, water is generated in both SS14 and SS15. Additionally, 1426–1427 cm^−1^ was the stretching vibration of the CO_3_^2−^ and C-O bonds due to the CO_2_ in the air [29]. The peak of SS14 here is stronger than that of SS15, proving that the carbonization is relatively strong, which corresponds to the peak strength of CaCO_3_ in the XRD. The strong peak at the characteristic line of zeolite at wave numbers of 1016–1022 cm^−1^ represented the non-uniform stretching vibration of Si-O bond, indicating that the alkali excited polymerization reaction [30]. The transition of the frequency band to a lower frequency in the range of 800–1200 cm^−1^ proved that the polymerization reaction was relatively intense [31,32], so the polymerization of SS15 is stronger than that of SS14. The wave numbers of 400–600 cm^−1^ and 600–800 cm^−1^ represented the bending vibration of Si-O and Si-O-Al, respectively [15]. The band at 456–460 cm^−1^ was attributed to the in-plane bending vibration of Si-O bond, and the band at 566–570 cm^−1^ was attributed to the contraction vibration of Si-O-Al [33]. The band at 730–733 cm^−1^ was attributed to the bending vibration of Si-O-Al formed after Al replaced Si [34].

### 4.3. Analysis of SEM and EDS

**Figure 14:**
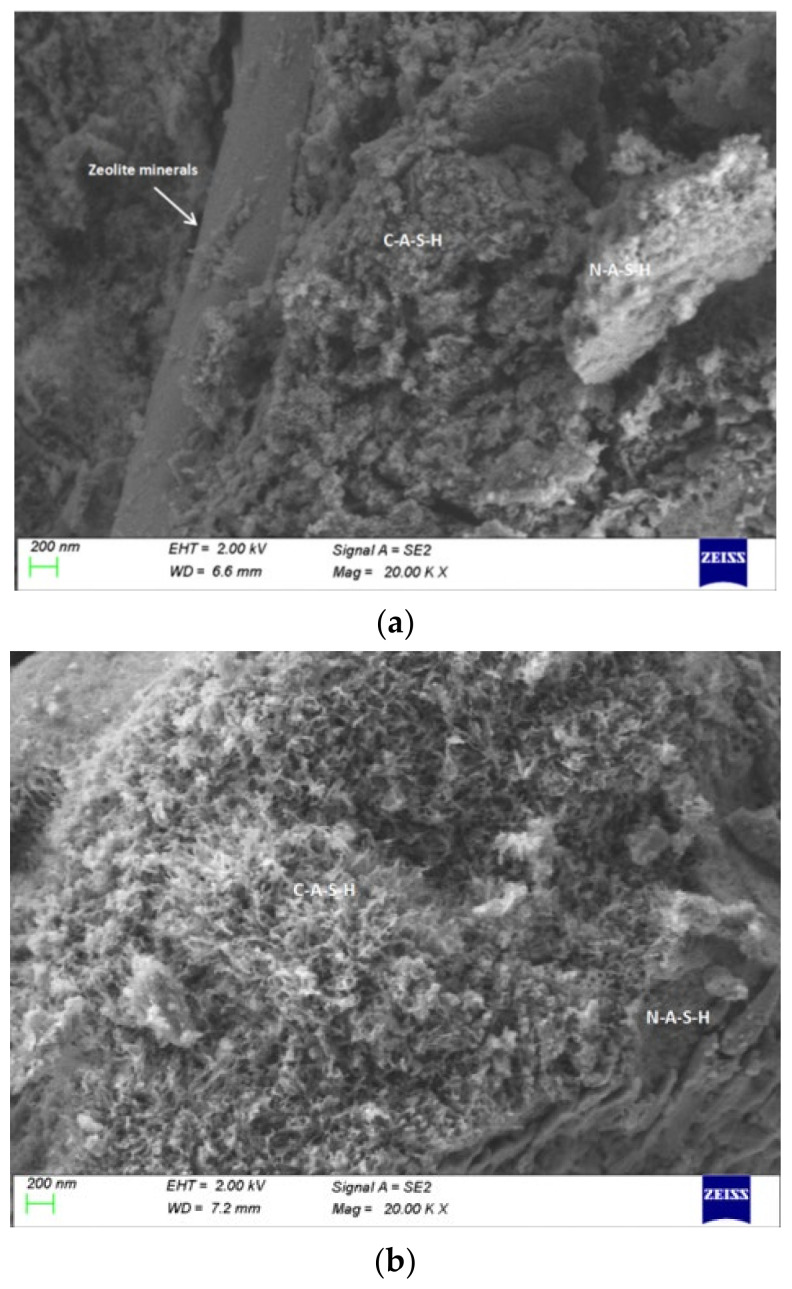
SEM images of geopolymers. (**a**) SS14 (**b**) SS15.

Figure 14 shows the SEM of geopolymers, demonstrating a considerable number of relatively dense gels in varying forms of flocs, clusters, nets, and blossoms were formed on the surface of geopolymers. The gels detected were mainly of N-A-S-H and C-A-S-H types. Obvious columnar zeolite products, C-A-S-H and N-A-S-H gel formation can be seen in SS14, and a large amount of C-A-S-H and N-A-S-H were formed in SS15. The reason why SS14 and SS15 can be excited to form geopolymer is that the activator contains sodium silicate. Silica tetrahedron is easy to polymerize in sodium silicate and can quickly form long chain silicate oligomer with the base material to form three-dimensional network silicoaluminate geopolymer [35].

**Figure 15:**
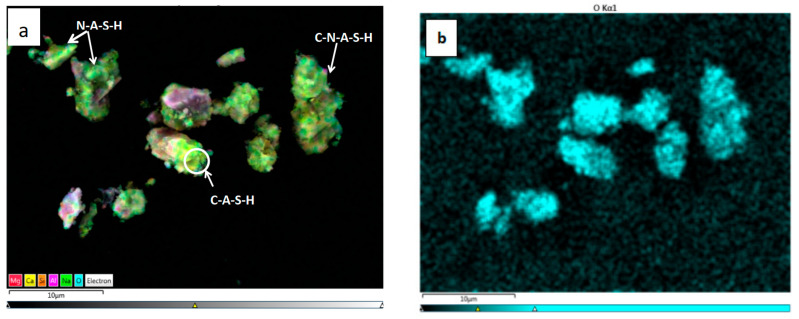

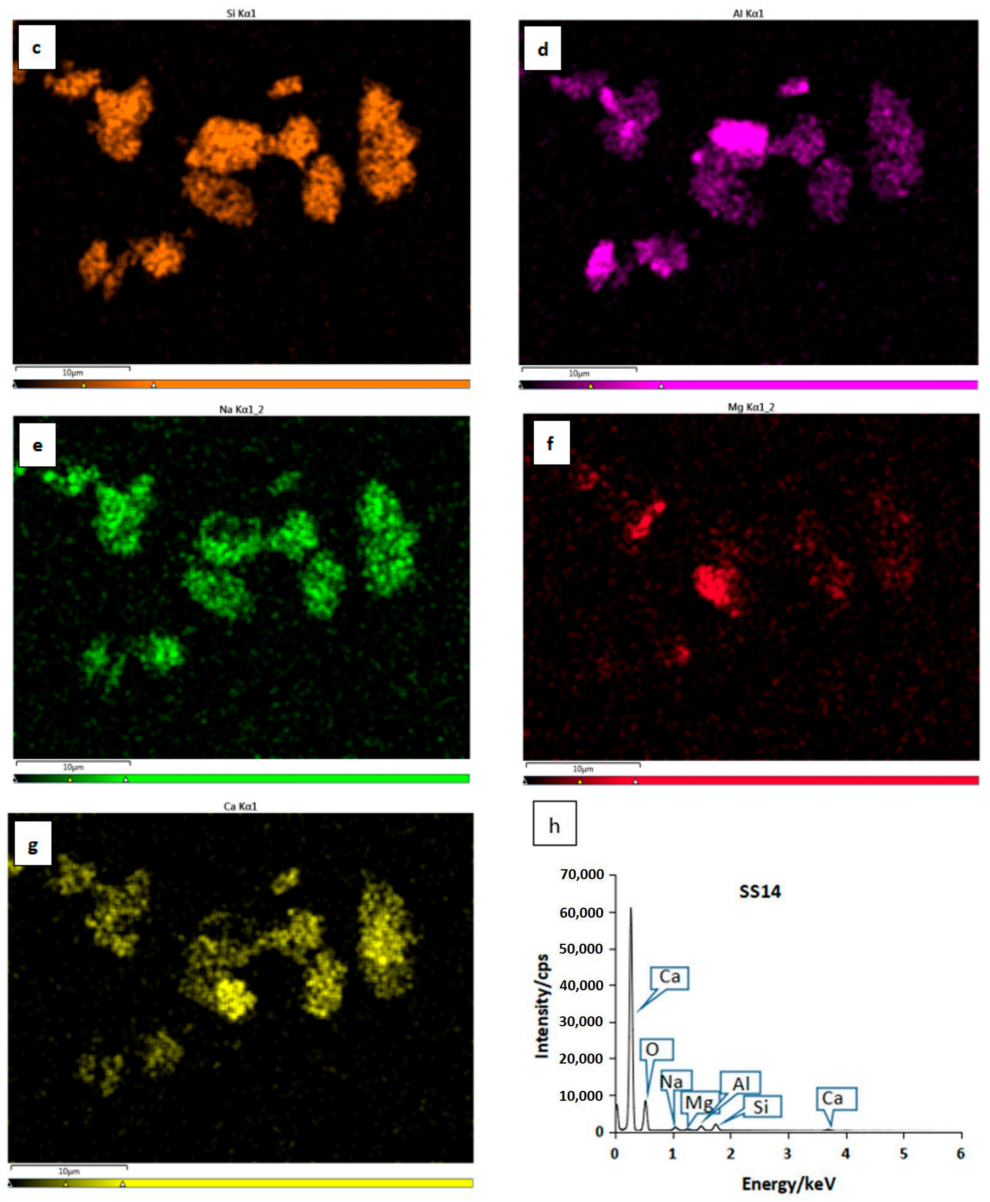
Element distribution diagram and EDS spectrum of SS14. (**a**) General distribution of major elements, (**b**) O element, (**c**) Si element, (**d**) Al element, (**e**) Na element, (**f**) Ca element, (**g**) Mg element, (**h**) EDS spectrum.

**Figure 16:**
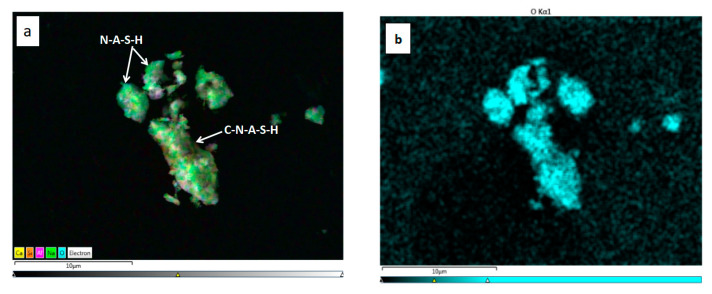

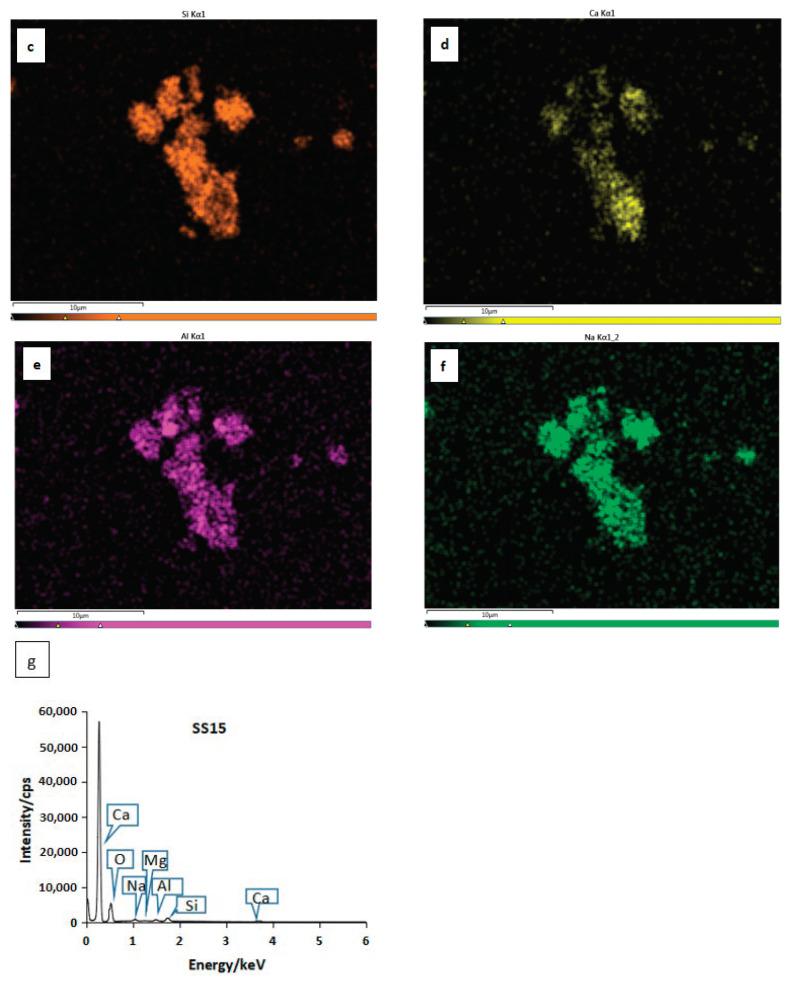
Element distribution diagram and EDS spectrum of SS15. (**a**) General distribution of major elements, (**b**) O element, (**c**) Si element, (**d**) Al element, (**e**) Na element, (**f**) Ca element, (**g**) EDS spectrum.

The surface scanning method was used in EDS to obtain the distribution of elements on the surface. Figure 15 and Figure 16 show the EDS analysis of geopolymers, revealing the main chemical elements of Ca, Si, Al, Na, and Mg in the two polymers. It can be seen from the overall distribution of elements in Figure 15a and Figure 16a depicting that C-A-S-H, N-A-S-H, and C-N-A-S-H overlapped and intertwined to cover an entire particle, indicating that these were hydration gels. The element diagram further shows that Si and Al elements were evenly distributed in each particle, and there were both overlaps and non-overlaps of Na and Ca elements. The overlapping parts were identified as C-N-A-S-H, the combination of C-A-S-H and N-A-S-H, and the non-overlapping parts were the corresponding hydration products.

Table 10 shows that the Ca/(Si + Al) of geopolymers were all less than 1.5. This is a feature of geopolymers [36]. An increase in Ca/Si, Si/Al, (Ca + Na)/(Si + Al) corresponded with an increase in the polymer, the compactness of structure, and the strength [37,38,39]. Therefore, SS14 and SS15 generated a high level of geopolymers with dense structure and high strength.

### 4.4. Mechanisms for Strength Development

From the foregoing discussions, it was found that the optimum alkali content and modulus was 18–20% and 0.6–0.8, respectively. The microscopic analyses of SS14 and SS15 showed that three types of gels and zeolite minerals were generated after alkali excitation. UCGP belongs to coal series metakaolin. Davidovits believed that after metakaolin was stimulated by alkali, the polymer formed was the main source of the strength development, and they proposed that the general molecular formula of the polymer was M_x_ [-(Si-O_2_) _z_-Al-O-] _n_·ωH_2_O [40]. M was an alkali metal element. The activator was NaOH and Na_2_SiO_3_, so the metal element was Na. The reaction formula is shown in reaction Equations (4) and (5) [41].

(4) $${\left({\mathrm{Si}}_{2}O_{5},{\;\mathrm{Al}}_{2}O_{2}\right)}_{n}+{\omega \mathrm{SiO}}_{2}+H_{2}O+\mathrm{NaOH}\to Na_{2n}{\left(\mathrm{OH}\right)}_{3}-\mathrm{Si}-O-\begin{array}{c} \mathrm{Al}\\ |\\ {\left(\mathrm{OH}\right)}_{2} \end{array}-O-\mathrm{Si}-{\left(\mathrm{OH}\right)}_{3}$$

(5) $$n{\left(\mathrm{OH}\right)}_{3}-\mathrm{Si}-O-\begin{array}{c} \mathrm{Al}\\ |\\ {\left(\mathrm{OH}\right)}_{2} \end{array}-O-\mathrm{Si}-{\left(\mathrm{OH}\right)}_{3}+\mathrm{NaOH}\to \mathrm{Na}{\left(-\begin{array}{c} \begin{array}{c} |\\ \mathrm{Si} \end{array}\\ |\\ O \end{array}-O-\begin{array}{c} \begin{array}{c} |\\ \mathrm{Al} \end{array}\\ |\\ O \end{array}-O-\begin{array}{c} \begin{array}{c} |\\ \mathrm{Si} \end{array}\\ |\\ O \end{array}-O-\right)}_{n}+{\mathrm{nH}}_{2}O$$

Because one of the base materials was BFS, the fracture and recombination of chemical bonds between Si, O, Al, and other elements also existed in the BFS after being activated by the alkaline solution. The strength came from the formation of C-S-H and C-A-S-H crystalline phases. The BFS reacted in an alkaline solution in Equations (6)–(8) [15,42].

(6) $$H_{3}{\mathrm{SiO}}_{4}^{-}+{\mathrm{Ca}}^{2+}+{\mathrm{OH}}^{-}\to {\left(\mathrm{CaO}\right)}_{x}\cdot {\left({\mathrm{SiO}}_{2}\right)}_{y}\cdot {\left(H_{2}O\right)}_{n}\left(C-S-H\;\mathrm{gel}\right)$$

(7) $$\begin{array}{c} {\mathrm{Ca}}^{2+}+H_{3}{\mathrm{SiO}}_{4}^{-}+{\mathrm{AlO}}_{2}^{-}+{\mathrm{OH}}^{-}+{\mathrm{CO}}_{3}^{2-}\\ \;\to \left(\mathrm{CaO}\right)\cdot {\left({\mathrm{Al}}_{2}O_{3}\right)}_{x}\cdot {\left({\mathrm{SiO}}_{2}\right)}_{y}\cdot {\left(H_{2}O\right)}_{n}+{\mathrm{CaCO}}_{3}+H_{2}O\;\left(C-A-S-H\;\mathrm{gel}\right) \end{array}$$

(8) $$\begin{array}{c} {\mathrm{Ca}}^{2+}+H_{3}{\mathrm{SiO}}_{4}^{-}+{\mathrm{AlO}}_{2}^{-}+{\mathrm{OH}}^{-}+{\mathrm{Na}}^{+}+{\mathrm{CO}}_{3}^{2-}\\ \;\to \left(\mathrm{CaO}\right){\left({\mathrm{Na}}_{2}O\right)}_{x}\cdot {\left({\mathrm{Al}}_{2}O_{3}\right)}_{y}{\left({\mathrm{SiO}}_{2}\right)}_{z}\cdot {\left(H_{2}O\right)}_{n}+{\mathrm{CaCO}}_{3}+H_{2}O\;\left(C-N-A-S-H\right)\;\mathrm{gel} \end{array}$$

This is in accordance with the fracture and vibration of Si-O-Si, Al-O-Al, and Al-O-Si chemical bonds in FTIR and the conclusion of the formation of N-A-S-H, C-(A)-S-H and C-N-A-S-H gels obtained by XRD, SEM and EDS. In addition, water was the mass transfer medium and reaction medium in the polymerization reaction and existed as bound water after solidification, which corresponded to the hydroxyl vibration in FTIR analysis. Therefore, the high strength of SS14 and SS15 specimens resulted from the generation of N-A-S-H, C-(A)-S-H gel, C-N-A-S-H gels, and zeolite minerals.

## 5. Conclusions

The compressive strength and bending strength of the coal-gangue-based geopolymer were evaluated and analyzed, and the microscopic analysis of the 28-day samples (SS14 and SS15) was carried out to elucidate the mechanisms for the strength development. The results of this study could be summarized as follows.

(1) The influencing factors affecting the strength development of the geopolymer in the high-low order of significance were BFS content, alkali content, and modulus.
(2) To maximize the strength development of the coal gangue base geopolymer, the optimum alkali content was 18–20%, the modulus was 0.6–0.8, and the corresponding compressive strength and flexural strength can reach more than 40 MPa and 5.9 MPa, respectively.
(3) The relationships of compressive strength or flexural strength to modulus conformed to a quadratic curve.
(4) The main reasons for the high strength of geopolymer were the strong reaction, dense structure, and the formation of three types of interlinked gels, namely N-A-S-H, C-A-S-H and C-N-A-S-H.

## Figures and Tables

**Table 1:** Chemical composition of UCGP (wt %).

Materials	SiO_2_	Al_2_O_3_	Fe_2_O_3_	CaO	K_2_O	TiO_2_	MgO	SO_3_	Na_2_O	Others
UCGP	54.2	41.6	0.98	0.55	0.91	0.96	0.10	0.02	0.46	0.22

**Table 2:** Basic physical and chemical properties of BFS powder.

Specific Surface Area/m^2^ × kg^−1^	Flow Ratio/%	7-Day Activity Index/%	28-Day Activity Index/%	Density/g × cm^−2^	Ignition Loss/%	Moisture Content/%
430	98	84.6	98.7	3.16	1.20	0.23
CaO	SiO_2_	Al_2_O_3_	SO_3_	Fe_2_O_3_	MgO	Others
33.5%	34.9%	16.7%	1.7%	1.05%	6.0%	6.15%

**Table 3:** Stage I: orthogonal experimental factors and levels.

Level	BFS Powder Content/%	Alkali Content/%	Modulus
	A	B	C
1	10	16	0.4
2	20	18	0.6
3	30	20	0.8
4	40	22	1.0

**Table 4:** Stage I: orthogonal design test.

Sample No.	SS1	SS2	SS3	SS4	SS5	SS6	SS7	SS8
BFS/%	10				20			
Alkali/%	16	18	20	22	16	18	20	22
Modulus	0.4	0.6	0.8	1.0	0.6	0.4	1.0	0.8
Sample No.	SS9	SS10	SS11	SS12	SS13	SS14	SS15	SS16
BFS/%	30				40			
Alkali/%	16	18	20	22	16	18	20	22
Modulus	0.8	1.0	0.4	0.6	1.0	0.8	0.6	0.4

**Table 5:** Stage II: the second stage of mixture design.

Sample No.	SS17	SS18	SS14	SS19	SS20	SS21	SS22	SS15	SS23
Alkali/%	18	18	18	18	18	18	20	20	20
Modulus	0.4	0.6	0.8	1.0	1.2	1.4	0.4	0.6	0.8
Sample No.	SS24	SS25	SS26	SS16	SS27	SS28	SS29	SS30	SS31
Alkali/%	20	20	20	22	22	22	22	22	22
Modulus	1.0	1.2	1.4	0.4	0.6	0.8	1.0	1.2	1.4

**Table 6:** Range and variance analyses of 3, 7, and 28-day compressive and flexural strengths.

Strength	Expectation Analysis Result and Optimum Combination of Influencing Factors	F_ratio_			F_critical_	Significance		
		A	B	C		A	B	C
3d compressive	A > B > C; A4B3C2	4.867	1.482	1.173	F_0.1_ = 3.29 F_0.05_ = 4.76 F_0.01_ = 9.78	**	-	-
3d flexural	A > B > C; A4B3C2	6.324	3.615	2.052		**	*	-
7d compressive	A > B > C; A4B3C2	5.667	1.787	0.870		**	-	-
7d flexural	A > B > C; A4B4C2	5.505	2.625	1.817		**	-	-
28d compressive	A > B > C; A4B2C2	4.152	1.606	0.358		*	-	-
28d flexural	A > B > C; A4B4C2/A4B4C1	5.749	3.526	1.918		**	*	-

Note: A-BFS powder content, B-alkali content, C-modulus. The F ratio is the sum of squared deviations of the factor divided by the sum of squared deviations of the error. Significance is determined by comparing F_ratio_ with F_critical_. F_0.05_ < F_ratio_ < F_0.01_ is significant **, F_0.1_ < F_ratio_ < F_0.05_ is generally significant *, F_ratio_ < F_0.1_ is not significant -.

**Table 7:** The ratio of 3-day and 7-day compressive strength of different geopolymers to 28-day compressive strength.

Geopolymer	F_(3,7)_/F_(28)_	Na_2_SiO_3_ Modulus					
		0.4	0.6	0.8	1.0	1.2	1.4
18% SS	F_3_/F_28_	0.41	0.51	0.57	0.65	0.21	0.04
	F_7_/F_28_	0.67	0.55	0.64	0.91	0.89	0.8
20% SS	F_3_/F_28_	0.51	0.64	0.57	0.55	0.40	0.28
	F_7_/F_28_	0.63	0.79	0.86	0.85	0.86	0.74
22% SS	F_3_/F_28_	0.54	0.60	0.57	0.49	0.49	0.5
	F_7_/F_28_	0.60	0.65	0.84	0.82	0.82	0.81

Note: F_3_, F_7_ and F_28_ are 3 days, 7 days and 28 days compressive strength, respectively.

**Table 8:** The ratio of 3-day and 7-day flexural strength of different geopolymers to 28-day compressive strength.

Geopolymer	F_(3,7)_/F_(28)_	Na_2_SiO_3_ Modulus					
		0.4	0.6	0.8	1.0	1.2	1.4
18% SS	F_3_/F_28_	0.78	0.8	0.54	0.59	0.38	0.06
	F_7_/F_28_	0.80	0.89	0.97	0.88	0.64	0.66
20% SS	F_3_/F_28_	0.63	0.92	0.82	0.86	0.72	0.71
	F_7_/F_28_	0.77	0.98	0.84	0.90	0.76	0.73
22% SS	F_3_/F_28_	0.79	0.62	0.72	0.76	0.66	0.50
	F_7_/F_28_	0.87	0.87	0.93	0.8	0.82	0.81

Note: F_3_, F_7_ and F_28_ are 3 days, 7 days, and 28 days flexural strength, respectively.

**Table 9:** Comparison of geopolymer strength and OPC.

Grade	Standard	Brittleness	Toughness
C25	SS22 (20% alkali, M0.4)		SS17 (18% alkali, M0.4)
C30	SS18, SS20 (18% alkali, M0.6, M1.2)	SS21, SS26, SS31 (18–22% alkali, M1.4)	SS16, SS27 (22% alkali M0.4–M0.6)
C35	SS19, SS24, SS28 (18–22% alkali, M1–M0.8)	SS25, SS29 (20–22% alkali, M1–1.2)	SS30 (22% alkali, M1.2)
C40	SS15, SS23 (20% alkali, M0.6–M0.8)		
C45		SS14 (18% alkali, M0.8)	

**Table 10:** The proportion of each element content by EDS test/%.

Element	O	Si	Ca	Al	Na	Mg	Ca/(Si + Al)	Ca/Si	Si/Al	(Ca + Na)/(Si + Al)
SS14	65.2	12.9	10.0	5.6	5.1	1.2	0.54	0.78	2.30	0.73
SS15	67.3	12.7	9.1	5.6	5.3	-	0.50	0.72	2.27	0.83

## Data Availability

Data obtained as described.
